# Patient characteristics and risk factors of early and late death in incident peritoneal dialysis patients

**DOI:** 10.1038/srep32359

**Published:** 2016-08-31

**Authors:** Xinhui Liu, Rong Huang, Haishan Wu, Juan Wu, Juan Wang, Xueqing Yu, Xiao Yang

**Affiliations:** 1 Department of Nephrology, The First Affiliated Hospital, Sun Yat-sen University, Guangzhou, Guangdong 510080, China; 2 Key Laboratory of Nephrology, Ministry of Health and Guangdong Province, Guangzhou, Guangdong 510080, China

## Abstract

This study was conducted to identify key patient characteristics and risk factors for peritoneal dialysis (PD) mortality in terms of different time-point of death occurrence. The incident PD patients from January 1, 2006 to December 31, 2013 in our PD center were recruited and followed up until December 31, 2015. Patients who died in the early period (the first 3 months) were older, had higher neutrophil to lymphocyte ratio (N/L), serum phosphorus, and uric acid level, and had lower diastolic pressure, hemoglobin, serum albumin, and calcium levels. After adjustment of gender, age, and PD inception, higher N/L level [hazard ratio (HR) 1.115, *P* = 0.006], higher phosphorus lever (HR 1.391, *P* < 0.001), lower hemoglobin level (HR 0.596, *P* < 0.001), and lower serum albumin level (HR 0.382, *P* = 0.017) were risk factors for early mortality. While, presence of diabetes (HR 1.627, *P* = 0.001), presence of cardiovascular disease (HR 1.847, *P* < 0.001) and lower serum albumin level (HR 0.720, *P* = 0.023) were risk factors for late mortality (over 24 months). In conclusion, patient characteristics and risk factors associated with early and late mortality in incident PD patients were different, which indicated specific management according to patient characteristics at the initiation of PD should be established to improve PD patient survival.

Peritoneal dialysis (PD) is a well-established modality of renal replacement therapy and is becoming more important in the management of patients with end-stage renal disease^1^. Due to government policies and its inherent advantages, PD has become wide spread in China in recent years. At the end of 2013, according to data from the Chinese Renal Data Registration System, the number of PD patients in mainland China was 46,000, accounting for 14.1% of total end-stage renal disease patients receiving dialysis^2^. Although patient survival and technique survival of PD patients have significantly improved during recent decades, reducing PD mortality remains a vital issue and is as important as facilitating the expansion of PD.

Recently, Lukowsky *et al*. found that the patterns and risk factors associated with early mortality (<3 months) in hemodialysis (HD) patients differ from those in later dialysis therapy periods using DaVita database^3^. Another study analyzed data from Fresenius Medical Care North America (FMCNA) health information system and found a more than twofold increase in the risk for death and hospitalization in the first 90 days of dialytic therapy^4^. This study also showed different relative risk of early (<2 weeks), incident (<90 days), and prevalent death (days 91–365) by risk factor. Most of previous studies have showed that PD had comparable or better patient survival than HD, but this benefit dissipated with time especially after 2 years of dialysis initiation^5^. Studies mentioned above suggested that risk factors of death occurred in different periods of dialysis were different and may contribute to high mortality of specific period. So, in order to improve PD patient survival, it is necessary to identify patient characteristics, cause of death, and risk factors of mortality in PD patients who died at different periods of treatment. Although many previous studies have investigated risk factors for mortality in PD patients, almost all of them have excluded patients who undertook PD therapy less than 3 months^6^,^7^. Additionally, none of previous study has investigated patterns and risk factors of early and late mortality in the same study population in PD patients. Therefore, we conducted this longitudinal cohort study to identify key patient characteristics and risk factors for PD mortality in terms of different time-point of death occurrence.

## Results

### Baseline Patient Characteristics

A total of 1778 incident PD patients who met the inclusion criteria were enrolled in this study. The mean (±SD) age was 47.4 ± 15.6 years; 59.5% of patients were men, and 25.3% of patients were diabetic. The primary cause of end-stage renal disease was chronic glomerulonephritis (59.7%) followed by diabetic nephropathy (22.5%) and hypertension (7.4%) (Table 1). Almost all of the patients received continuous ambulatory peritoneal dialysis treatment, except two patients who used automated peritoneal dialysis. Conventional PD solutions (Dianeal 1.5%, 2.5% or 4.25% dextrose; Baxter Healthcare, Guangzhou, China), Y-sets, and twin-bag systems were utilized in all PD patients. The management of the PD patients was implemented based on the guidelines and recommendations from the International Society for Peritoneal Dialysis^8^.

### Outcome

**Figure 1:**
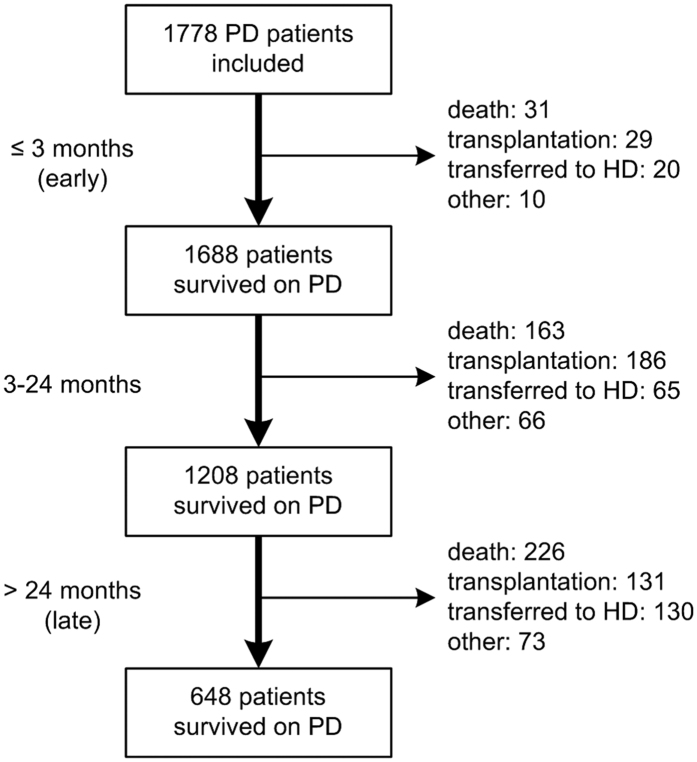
Patient distribution over three follow-up periods. HD, hemodialysis; PD, peritoneal dialysis.

**Figure 2:**
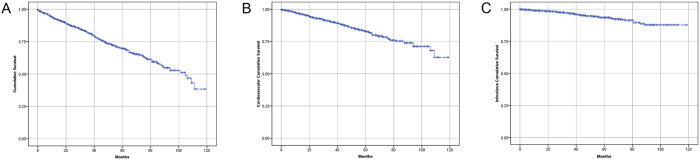
Survival curves of all-cause, cardiovascular, and infectious death. Cumulative mortality curves for (**A**) all-cause mortality, (**B**) cardiovascular mortality, and (**C**) infectious mortality.

**Figure 3:**
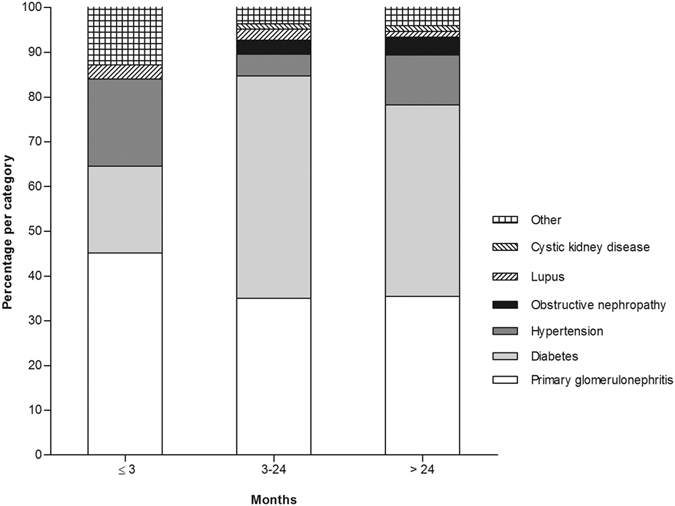
Primary cause of end-stage renal disease for patients who died during different follow-up periods of PD treatment.

**Figure 4:**
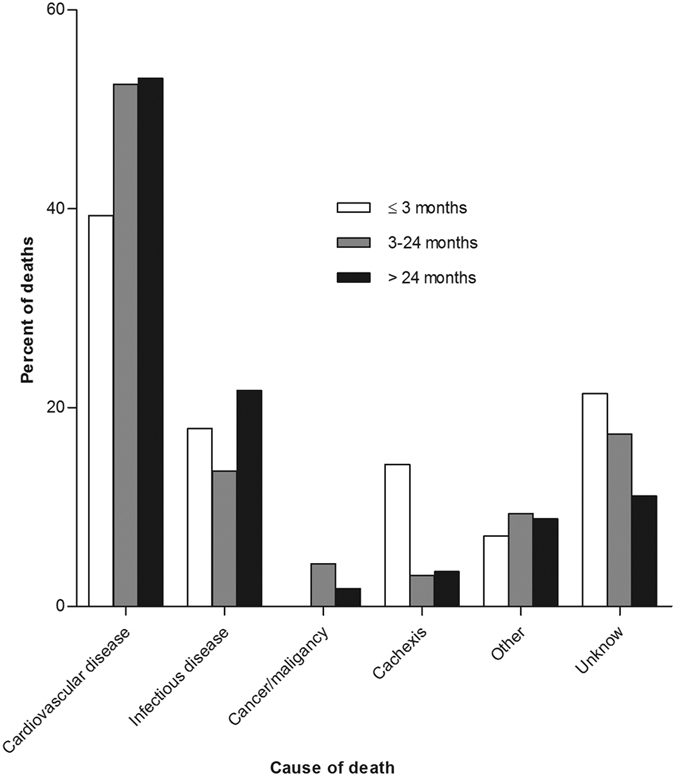
Causes of death for patients who died during different follow-up periods of PD treatment.

The median follow-up period was 35 months (interquartile range 17 to 56 months). By the end of this study, 420 (23.6%) patients had died, 346 (19.5%) patients had received kidney transplantation, 215 (12.1%) patients had transferred to HD, 79 (4.4%) patients had transferred to other PD centers, 48 (2.7%) patients had been lost to follow-up, 18 (1.0%) patients had declined additional treatment, and 4 (0.2%) patients had stopped PD treatment due to kidney function restoration; the remaining 648 (36.4%) patients were still followed at our PD center. Of 420 died patients, 31 (7.4%) deaths occurred in the first 3 months (early) and 226 (53.8%) deaths occurred over 24 months (late). Figure 1 shows the distribution of death, renal transplantation, and transfer to HD during the three predefined periods of follow-up. The estimated mean survival time of all-cause death, cardiovascular death, and infectious death was 83.3 months, 98.3 months, and 111.1 months, respectively by Kaplan–Meier analyses (Fig. 2). Compared with patients who survived the first 3 months, patients who died within the first 3 months were older, had higher comorbidity score, neutrophil to lymphocyte ratio (N/L), and serum phosphorus levels, had lower measured glomerular filtration rate (mGFR), diastolic pressure, hemoglobin, albumin, and serum calcium levels. The comparison of patient characteristics at the landmark of 24 months was similar with above comparison at the landmark of 3 months. But beyond that, patients who died during 3 to 24 months were more likely to be diabetic and have cardiovascular disease, had lower 24 h urine output, intact parathyroid hormone (iPTH), and uric acid levels, compared with patients who survived the first 24 months (Table 2). Baseline characteristics of the patients who died within different follow-up periods are shown in Supplemental Table S1. Patients who died in the early period were older, had higher N/L, serum phosphorus, iPTH, and uric acid level, and had lower diastolic pressure, mGFR, hemoglobin, serum albumin, and calcium levels (see Supplemental Table S1). Figure 3 shows the primary cause of end-stage renal disease among patients who died during each of a priori selected periods of follow-up. Chronic glomerulonephritis was more common among patients dying early compared with those who died late (45.2% *versus* 35.4%, *P* = 0.290), whereas diabetic nephrology was more common among patients dying late (42.9% *versus* 19.4%, *P* = 0.012). Figure 4 shows the causes of death during different follow-up periods. Cardiovascular death was more common (53.1%) among late death, compared with early death (39.3%) (*P* = 0.066).

### Risk factors for mortality

After adjusted gender, age, and PD inception, the presence of diabetes, cardiovascular disease, higher N/L level, and lower hemoglobin level were risk factors for both all-cause and cardiovascular mortality. For infectious mortality, only presence of cardiovascular disease [hazard ratio (HR) 2.367, 95% confidence interval (CI) 1.391–4.029, *P* = 0.001] and lower serum albumin level (HR 0.593, 95% CI 0.367–0.957, *P* = 0.032) showed statistical significance in multivariate Cox model. In addition, lower 24 h urine output was only a risk factor for all-cause mortality (HR 0.978, 95% CI 0.958–0.998, *P* = 0.031) in our analyses (Table 3). Risk factors for death occurred on the three predefined periods of follow-up were summarized in Table 4. After adjustment of gender, age, and PD inception, higher N/L level (HR 1.115, 95% CI 1.031–1.205, *P* = 0.006), higher phosphorus level (HR 1.391, 95% CI 1.164–1.663, *P* < 0.001), lower hemoglobin level (HR 0.596, 95% CI 0.483–0.737, *P* < 0.001), and lower serum albumin level (HR 0.382, 95% CI 0.173–0.843, *P* = 0.017) were risk factors for early mortality. While, presence of diabetes (HR 1.627, 95% CI 1.213–2.181, *P* = 0.001), presence of cardiovascular disease (HR 1.847, 95% CI 1.378–2.475, *P* < 0.001) and lower serum albumin level (HR 0.720, 95% CI 0.543–0.956, *P* = 0.023) were risk factors for late mortality. The *P* value of 24 h urine output and hemoglobin in multivariate model for late mortality were in the margin of statistical significance (*P* = 0.064, and 0.077, respectively).

## Discussion

In the present study, we identified patient characteristics and risk factors of mortality in PD patients who died at different periods of treatment. We found that patient characteristics, primary cause of end-stage renal disease, and cause of death were quite distinct among PD patients died within different periods of follow-up, and the risk factor pattern altered with PD duration.

Patients who died early, in our study, were older, have higher comorbidity score and N/L level, and have significant symptoms of uremia (anemia, malnutrition, hypocalcemia, hyperphosphatemia, and hyperuricemia). These clinical characteristics may contribute to early death and distinct risk factor pattern of early and late mortality. Hyperphosphatemia is independently associated with an increased risk of death among dialysis patients^9^. Our study revealed that higher serum phosphorus level was associated with mortality only in early died PD patients after multivariate adjusted. It was found that serum phosphorus level was significant different between patients who died early and those who survived the first 3 months, while this difference was not significant between patients who died during 3 to 24 months and those who survived the first 24 months (Table 2). Results above may indicate more serious loss of nephrons in patients dying early, actually these patients showed more overt uremia performance. The association of timing of PD initiation with survival is controversial^10^,^11^,^12^. A prospective cohort study based on Hong Kong Peritoneal Dialysis Study Group found that patients who refuse timely start of dialysis have worse overall outcome at one year after the offer of dialysis, compared with elective starters who were electively initiated on PD when GFR reached 10 ml/min per 1.73m^2^ or below^13^. In our study, the significant symptoms of uremia and poor residual renal function (RRF) indicated that these patients conducted late PD initiation, which may be associated with early death occurrence, especially for elderly end-stage renal disease patients. Previous study has shown that N/L, a marker of inflammation, was a strong predictor of mortality in PD patients^14^. However, in our study, higher N/L level was only a risk factor for early death in multivariate model. This result may be related to advanced age and lowest serum albumin level of early died patients, as aging, malnutrition, and inflammation appear to be interrelated, each additionally contributing to mortality in these patients^15^,^16^. In addition, infection is a common cause of mortality in patients with end-stage renal disease^17^,^18^. Therefore, higher N/L level may also imply that infective component prior to PD inception plays a role in early mortality.

In our study, 194 patients died during the first 24 months of PD treatment, 31 of them occurred in the initial 3 months (16.0%), which was lower compared with previous study in incident HD patients (30.0%) in DaVita clinic system^3^. We found that fatal cardiovascular disease were more frequent among patients who survived longer than 3 months, whereas previous studies in HD patients show that cardiovascular death was more frequent in early death^3^,^19^. The clinical significance of this difference is unclear. In addition, among clinical predictors, presence of diabetes was exceptionally not associated with early mortality even in univariate model. This result were similar with previous studies in HD patients reported by Bradbury *et al*.^19^ and Lukowsky *et al*.^3^, which also showed paradoxical association between diabetes and mortality in the first months. The possible reason may be that compared with nondiabetics or non-cardiovascular disease patients, diabetics or cardiovascular disease patients were more likely to see a physician and better prepared for the transitional period of early dialysis therapy. However, with dialysis prolongation, the existence of diabetic or cardiovascular disease gradually manifested its adverse effect on patient survival. Not surprisingly, RRF was an independent risk factor for mortality in those who survived longer than 3 months. In line with our results, a study from USA found RRF was an important predictor of 1-year mortality in chronic PD patients by analyzing a national database—the End-Stage Renal Disease Core Indicators Project^20^. Another study from Korea demonstrated low RRF was significantly correlated with mortality of those who maintained PD for more than 2 years^21^. The plausible explanations for the importance of RRF in predicting mortality include reducing volume overload, reducing use of hypertonic dialysis solutions, increasing renal clearance of larger molecular weight molecules^22^,^23^. These protective effects will be more obvious with PD duration, so the predictive effect of RRF in early death was not significant in our study.

There are some limitations of present study. First, our study was a single center study, and therefore, center-specific effects cannot be excluded. Second, given the retrospective nature of our study, we established associations but not causal relationships. Third, due to restriction of sample size, we did not adjust all factors associated with mortality. So, the effect of residual confounding cannot be eliminated completely.

In conclusion, we found distinct patient characteristics and risk factors for early and late mortality in PD patients. These findings suggest that special attention be paid to special risk factors with respect to PD vintage, which would be benefit to better patient survival on PD treatment.

## Methods

### Patients

We studied all incident patients who used PD as their first renal replacement therapy (RRT) modality and who were followed up at the PD center of The First Affiliated Hospital, Sun Yat-sen University, Guangzhou, China from January 1, 2006, to December 31, 2013. The patients who were less than 18 years at the start of PD, catheterized in other hospitals, transferred from permanent HD (≥3 months) or failed renal transplantation were excluded in this study. The study was conducted in compliance with the ethical principles of the Helsinki Declaration (http://www.wma.net/en/30publications/10policies/b3/index.html) and approved by the Human Ethics Committees of Sun Yat-sen University. As a part of a larger cohort study, written informed consent was obtained from all participants. All patients were followed up until cessation of PD, death or on December 31, 2015.

### Data Collection

Baseline demographic data included age, gender, primary cause of end-stage renal disease and presence of diabetes and cardiovascular disease. Cardiovascular disease was defined as a past history of or current myocardial infarction, angina, peripheral vascular disease or cerebrovascular disease. Clinical and biochemical data at the initiation of PD included body mass index (BMI), blood pressure, residual urine volume, hemoglobin, serum albumin, serum creatinine, blood urea nitrogen, total cholesterol, triglycerides, serum calcium, phosphorus, intact parathyroid hormone (iPTH) and uric acid. All data were obtained at the initiation of PD treatment. The comorbidity score was determined according to the Charlson Comorbidity Index, which is one of the most commonly used comorbidity models^24^.

### Statistical analyses

To identify patient characteristics and risk factors for early and late death during PD treatment, we divided the follow-up time into three survival periods: the first 3 months of PD therapy (early); 3 to 24 months; over 24 months (late). We compared demographic, clinical and laboratory data of patients who were dead or alive at the time-point of follow-up, and patients who died within predefined periods of follow-up. Results were expressed as frequencies and percentages for categorical variables, means and standard deviations for normally distributed continuous variables and medians and interquartile ranges for continuous variables not normally distributed. Chi-squared, *t*-tests or one-way ANOVA, and Mann-Whitney tests or Kruskal-Wallis tests were used to test for differences in categorical or continuous factors among different groups. Survival times were estimated from Kaplan–Meier curves. Cause-specific Cox proportional hazards models were used to identify risk factors of all-cause, cardiovascular, and infectious mortality in all patients. Cox proportional hazards models were used to calculate hazard ratios of death at different periods of follow-up. The censored data included switching to HD, renal transplantation, moving to another center, declining additional treatment, loss to follow-up or still at our PD center on December 31, 2015. For each selected period, deaths after the period were also censored. In the cause-specific Cox model, gender, age, PD inception, and covariates with *P* < 0.05 in the univariate analysis were included in multivariate Cox proportional hazards regression. In the analysis of risk factors for mortality at different time periods, the variables included in the univariate Cox model were gender, age, PD inception, previous diabetes, previous cardiovascular disease, 24 h urine volume, neutrophil to lymphocyte ratio (N/L), hemoglobin, serum albumin, and phosphorus. Gender, age, PD inception, and covariates with *P* values < 0.2 in the univariate Cox analyses were used for multivariate Cox proportional hazards regression. The results were expressed as the hazard ratio (HR) and 95% confidence interval (CI). All descriptive and multivariate analyses were conducted using SPSS version 16.0 (SPSS Inc., Chicago, IL, USA). A value of *P* < 0.05 was considered statistically significant.

## Additional Information

**How to cite this article**: Liu, X. *et al*. Patient characteristics and risk factors of early and late death in incident peritoneal dialysis patients. *Sci. Rep.* **6**, 32359; doi: 10.1038/srep32359 (2016).

## Supplementary Material

Supplementary Information

## Figures and Tables

**Table 1:** Demographic characteristics of all patients included in present study.

Variable	Analysis cohort (n = 1778)
Age (years)	47.4 ± 15.6
Male gender (%)	1058 (59.5)
PD inception	
2006–2009 (%)	841 (47.3)
2010–2013 (%)	937 (52.7)
Body mass index (kg/m^2^)	21.5 ± 3.0
Diabetes (%)	449 (25.3)
CVD (%)	693 (39.0)
Primary kidney disease	
Chronic glomerulonephritis (%)	1062 (59.7)
Diabetic nephropathy (%)	400 (22.5)
Hypertension (%)	131 (7.4)
Other (%)	185 (10.4)
Comorbidity score	3 (2–5)
24 h urine output (ml)	1000 (600–1500)
mGFR (ml/min per 1.73 m^2^)	6.4 (5.0–8.5)
Systolic pressure (mmHg)	136 ± 20
Diastolic pressure (mmHg)	84 ± 14
Hemoglobin (g/dl)	10.1 ± 2.1
WBC (10^9^/L)	6.6 (5.3–8.1)
N/L	2.87 (2.14–3.92)
Albumin (g/dl)	3.7 ± 0.5
ALT (U/L)	15 (10–22)
AST (U/L)	19 (15–25)
ALP (U/L)	70 (56–89)
Calcium (mg/dl)	9.02 ± 0.93
Phosphorus (mg/dl)	4.43 ± 1.41
iPTH (pg/ml)	224 (99–402)
Urea nitrogen (mg/dl)	95 (76–120)
Creatinine (mg/dl)	7.9 (6.3–10.1)
Total cholesterol (mg/dl)	189 (162–224)
Triglyceride (mg/dl)	125 (89–180)
HDL (mg/dl)	45.2 (36.3–56.5)
LDL (mg/dl)	109.4 (87.0–133.0)
Uric acid (mg/dl)	7.14 ± 1.98

Abbreviations: ALP, alkaline phosphatase; ALT, alanine aminotransferase; AST, aspartate aminotransferase; CVD, cardiovascular disease; HDL, high density lipoprotein; iPTH, intact parathyroid hormone; LDL, low density lipoprotein; mGFR, measured glomerular filtration rate; N/L, neutrophil to lymphocyte ratio; PD, peritoneal dialysis; WBC, white blood cell.

**Table 2:** Demographic characteristics of those who are alive or dead at different landmark.

Variable	Died less than 3 months on PD (n = 31)	Survived the first 3 months on PD (n = 1688)	*P value*	Died during 3 to 24 months on PD (n = 163)	Survived the first 24 months on PD (n = 1208)	*P value*
Age (years)	64.9 ± 17.0	47.2 ± 15.4	**<0.001**	61.3 ± 14.2	47.0 ± 14.6	**<0.001**
Male gender (%)	13 (41.9)	1006 (59.6)	**0.047**	86 (52.8)	700 (57.9)	0.209
Body mass index (kg/m^2^)	22.4 ± 3.1	21.5 ± 3.1	0.296	21.8 ± 3.4	21.5 ± 3.0	0.242
Diabetes (%)	8 (25.8)	429 (25.4)	0.960	89 (54.6)	296 (24.5)	**<0.001**
CVD (%)	16 (51.6)	663 (39.3)	0.164	112 (68.7)	451 (37.3)	**<0.001**
Comorbidity score	6 (4–8)	3 (2–5)	**<0.001**	6 (4–7)	3 (2–5)	**<0.001**
24 h urine output (ml)	825 (505–1288)	1000 (600–1500)	0.324	700 (300–1100)	1000 (600–1500)	**<0.001**
mGFR (ml/min per 1.73 m^2^)	5.4 (4.2–6.9)	6.5 (5.0–8.5)	**0.011**	7.2 (5.2–9.5)	6.5 (5.1–8.5)	**0.034**
Systolic pressure (mmHg)	139 ± 20	136 ± 20	0.427	135 ± 21	136 ± 20	0.497
Diastolic pressure (mmHg)	76 ± 14	84 ± 14	**0.003**	76 ± 15	84 ± 14	**<0.001**
Hemoglobin (g/dl)	7.6 ± 2.0	10.2 ± 2.0	**<0.001**	9.5 ± 2.1	10.3 ± 2.0	**<0.001**
WBC (10^9^/L)	7.5 (6.2–12.0)	6.6 (5.3–8.0)	**0.001**	7.4 (6.3–8.9)	6.5 (5.3–8.0)	**<0.001**
N/L	4.63 (2.45–9.91)	2.83 (2.13–3.85)	**<0.001**	3.48 (2.40–4.75)	2.76 (2.12–3.72)	**<0.001**
Albumin (g/dl)	3.0 ± 0.7	3.7 ± 0.5	**<0.001**	3.4 ± 0.5	3.7 ± 0.5	**<0.001**
ALT (U/L)	22 (16–28)	14 (10–22)	**<0.001**	14 (10–19)	14 (10–22)	0.366
AST (U/L)	13 (8–22)	19 (15–25)	**0.001**	17 (15–23)	19 (15–25)	0.147
ALP (U/L)	90 (56–120)	70 (56–88)	0.060	77 (62–102)	70 (56–88)	**0.001**
Calcium (mg/dl)	8.25 ± 0.98	9.05 ± 0.92	**<0.001**	8.90 ± 0.97	9.06 ± 0.92	**0.039**
Phosphorus (mg/dl)	5.51 ± 1.95	4.37 ± 1.35	**0.004**	4.44 ± 1.80	4.30 ± 1.29	0.354
iPTH (pg/ml)	270 (145–520)	221 (97–401)	0.101	161 (49–360)	240 (105–409)	**0.001**
Urea nitrogen (mg/dl)	136 (106–177)	94 (76–118)	**<0.001**	89 (67–113)	94 (76–116)	0.067
Creatinine (mg/dl)	8.4 (6.3–9.6)	7.9 (6.3–10.0)	0.830	6.6 (5.5–8.7)	7.7 (6.3–9.7)	**<0.001**
Total cholesterol (mg/dl)	178 (152–230)	193 (162–224)	0.413	197 (155–241)	193 (166–224)	0.529
Triglyceride (mg/dl)	127 (97–244)	124 (89–178)	0.166	140 (99–223)	123 (88–174)	**0.001**
HDL (mg/dl)	33.3 (24.0–40.6)	45.6 (37.1–56.7)	**<0.001**	41.4 (32.3–52.2)	46.4 (37.9–57.2)	**<0.001**
LDL (mg/dl)	104.0 (91.3–138.4)	109.8 (87.0–133.4)	0.985	111.4 (82.1–146.8)	110.2 (88.2–133.8)	0.479
Uric acid (mg/dl)	7.95 ± 2.70	7.10 ± 1.94	0.105	6.71 ± 1.65	7.10 ± 2.02	**0.021**

Abbreviations: ALP, alkaline phosphatase; ALT, alanine aminotransferase; AST, aspartate aminotransferase; CVD, cardiovascular disease; HDL, high density lipoprotein; iPTH, intact parathyroid hormone; LDL, low density lipoprotein; mGFR, measured glomerular filtration rate; N/L, neutrophil to lymphocyte ratio; PD, peritoneal dialysis; WBC, white blood cell. The boldface indicated that *P* values less than 0.05 are considered statistically significant.

**Table 3:** Risk factors for all-cause, cardiovascular, and infectious mortality.

Variable	All-cause death (n = 420)		Cardiovascular death (n = 216)		Infectious death (n = 76)	
	Multivariate (HR, 95% CI)	*P value*	Multivariate (HR, 95% CI)	*P value*	Multivariate (HR, 95% CI)	*P value*
Gender (male *versus* female)	1.011 (0.825–1.238)	0.919	1.038 (0.784–1.374)	0.793	0.946 (0.588–1.521)	0.818
Age (per 5 years)	1.280 (1.226–1.335)	**<0.001**	1.321 (1.243–1.403)	**<0.001**	1.277 (1.153–1.414)	**<0.001**
Dialysis inception (2010–2013 *versus* 2006–2009)	0.648 (0.519–0.810)	**<0.001**	0.609 (0.447–0.829)	**0.002**	0.780 (0.464–1.313)	0.350
Diabetes (yes *versus* no)	1.398 (1.130–1.730)	**0.002**	1.525 (1.140–2.041)	**0.005**	1.445 (0.881–2.370)	0.144
CVD (yes *versus* no)	1.859 (1.488–2.321)	**<0.001**	2.064 (1.515–2.812)	**<0.001**	2.367 (1.391–4.029)	**0.001**
24 h urine output (per 100 ml)	0.978 (0.958–0.998)	**0.031**	0.990 (0.963–1.018)	0.488	0.963 (0.916–1.011)	0.132
N/L	1.067 (1.030–1.105)	**<0.001**	1.072 (1.022–1.124)	**0.005**	1.057 (0.971–1.150)	0.199
Hemoglobin (per g/dl)	0.883 (0.837–0.933)	**<0.001**	0.869 (0.806–0.938)	**<0.001**	0.898 (0.791–1.020)	0.097
Albumin (per g/dl)	0.677 (0.553–0.830)	**<0.001**	0.810 (0.609–1.077)	0.147	0.593 (0.367–0.957)	**0.032**

Abbreviations: CI, confidence interval; CVD, cardiovascular disease; HR, hazard ratio; N/L, neutrophil to lymphocyte ratio.

The boldface indicated that *P* values less than 0.05 are considered statistically significant.

**Table 4:** Risk factors for mortality at different time periods.

Variable	≤3 months (n = 31)				3 to 24 months (n = 163)				>24 months (n = 226)			
	Univariate (HR, 95% CI)	*P value*	Multivariate (HR, 95% CI)	*P value*	Univariate (HR, 95% CI)	*P value*	Multivariate (HR, 95% CI)	*P value*	Univariate (HR, 95% CI)	*P value*	Multivariate (HR, 95% CI)	*P value*
Gender (male *versus* female)	0.489 (0.240–0.998)	0.049	0.572 (0.267–1.227)	0.151	0.786 (0.578–1.069)	0.125	0.979 (0.706–1.357)	0.899	1.066 (0.819–1.387)	0.634	1.134 (0.859–1.497)	0.373
Age (per 5 years)	1.477 (1.295–1.683)	<0.001	1.369 (1.199–1.562)	**<0.001**	1.398 (1.320–1.482)	<0.001	1.267 (1.182–1.357)	**<0.001**	1.340 (1.274–1.410)	<0.001	1.260 (1.187–1.336)	**<0.001**
Dialysis inception (2010–2013 *versus* 2006–2009)	0.564 (0.274–1.162)	0.120	1.082 (0.483–2.426)	0.848	0.542 (0.396–0.742)	<0.001	0.652 (0.463–0.920)	**0.015**	0.669 (0.498–0.900)	0.008	0.699 (0.511–0.957)	**0.025**
Diabetes (yes *versus* no)	1.029 (0.460–2.300)	0.945			3.533 (2.596–4.809)	<0.001	1.492 (1.055–2.109)	**0.024**	3.259 (2.505–4.242)	<0.001	1.627 (1.213–2.181)	**0.001**
CVD (yes *versus* no)	1.668 (0.825–3.373)	0.155			3.504 (2.516–4.879)	<0.001	2.033 (1.390–2.973)	**<0.001**	2.821 (2.162–3.680)	<0.001	1.847 (1.378–2.475)	**<0.001**
24 h urine volume (per 100 ml)	0.965 (0.902–1.031)	0.289			0.920 (0.892–0.949)	<0.001	0.966 (0.934–0.999)	**0.046**	0.955 (0.931–0.980)	<0.001	0.974 (0.947–1.002)	0.064
N/L	1.215 (1.155–1.278)	<0.001	1.115 (1.031–1.205)	**0.006**	1.125 (1.079–1.173)	<0.001	1.053 (0.997–1.112)	0.066	1.082 (1.017–1.152)	0.013	1.023 (0.959–1.092)	0.488
Phosphorus (per mg/dl)	1.392 (1.199–1.616)	<0.001	1.391 (1.164–1.663)	**<0.001**	1.048 (0.939–1.171)	0.400			1.002 (0.908–1.106)	0.964		
Hemoglobin (per g/dl)	0.534 (0.443–0.644)	<0.001	0.596 (0.483–0.737)	**<0.001**	0.818 (0.757–0.883)	<0.001	0.860 (0.784–0.943)	**0.001**	0.941 (0.882–1.004)	0.064	0.937 (0.872–1.007)	0.077
Albumin (per g/dl)	0.161 (0.095–0.276)	<0.001	0.382 (0.173–0.843)	**0.017**	0.349 (0.266–0.457)	<0.001	0.719 (0.519–0.996)	**0.048**	0.439 (0.340–0.567)	<0.001	0.720 (0.543–0.956)	**0.023**

Abbreviations: CI, confidence interval; CVD, cardiovascular disease; HR, hazard ratio; N/L, neutrophil to lymphocyte ratio.

The boldface indicated that *P* values less than 0.05 in multivariate model are considered statistically significant.
