# Supplementary material for: Patient characteristics and risk factors of early and late death in incident peritoneal dialysis patients
Source: Sci Rep. 2016 Aug 31;6:32359. doi: 10.1038/srep32359 (PMC5006021; doi:10.1038/srep32359)
Supplement: Supplementary Information [file srep32359-s1.pdf]

Patient characteristics and risk factors of early and late death in incident peritoneal dialysis patients

Xinhui Liu[a,b], Rong Huang[a,b], Haishan Wu[a,b], Juan Wu[a,b], Juan Wang[a,b], Xueqing Yu[a,b], Xiao Yang[a,b,*]

[a]Department of Nephrology, The First Affiliated Hospital, Sun Yat-sen University, Guangzhou, Guangdong 510080, China

[b]Key Laboratory of Nephrology, Ministry of Health and Guangdong Province, Guangzhou, Guangdong 510080, China

Correspondence to: Prof. Xiao Yang, PhD & MD

Full address: Department of Nephrology, The First Affiliated Hospital, Sun Yat-sen University, Guangzhou, Guangdong 510080, China

Tel number: 86-20-87755766-8843

Fax number: 86-20-87766335

E-mail: yangxsysu@126.com

Supplemental Table S1. Demographic characteristics for those who died at different times of follow-up period

Variable	Death ≤ 3 months (n = 31)	Death 3 to 24 months (n = 163)	Death > 24 months (n = 226)	*P value*
Age (years)	64.9 ± 17.0	61.3 ± 14.2	57.3 ± 13.5	**0.002**
Male gender (%)	13 (41.9)	86 (52.8)	127 (56.2)	0.309
Body mass index (kg/m^2)	22.4 ± 3.1	21.8 ± 3.4	21.8 ± 2.9	0.789
Diabetes (%)	8 (25.8)	89 (54.6)	107 (47.3)	**0.011**
CVD (%)	16 (51.6)	112 (68.7)	121 (53.5)	**0.007**
Comorbidity score	6 (4-8)	6 (4-7)	5 (4-6)	**0.048**
24h urine volume (ml)	825 (505-1288)	700 (300-1100)	800 (465-1125)	0.174
mGFR (ml/min per 1.73 m^2)	5.4 (4.2-6.9)	7.2 (5.2-9.5)	7.1 (5.1-9.1)	**0.007**
Systolic pressure (mmHg)	139 ± 20	135 ± 21	138 ± 23	0.340
Diastolic pressure (mmHg)	76 ± 14	76 ± 15	81 ± 16	**0.007**
Hemoglobin (g/dl)	7.6 ± 2.0	9.5 ± 2.1	10.1 ± 1.9	**<0.001**
WBC (10^9/L)	7.5 (6.2-12.0)	7.4 (6.3-8.9)	7.0 (5.6-8.5)	**0.014**
N/L	4.63 (2.45-9.91)	3.48 (2.40-4.75)	2.85 (2.13-3.97)	**<0.001**
Albumin (g/dl)	3.0 ± 0.7	3.4 ± 0.5	3.5 ± 0.5	**<0.001**
ALT (U/L)	22 (16-28)	14 (10-19)	15 (9-22)	**<0.001**
AST (U/L)	13 (8-22)	17 (15-23)	18 (14-24)	**0.007**
ALP (U/L)	90 (56-120)	77 (62-102)	83 (61-106)	0.573
Calcium (mg/dl)	8.25 ± 0.98	8.90 ± 0.97	9.03 ± 0.91	**<0.001**

Phosphorus (mg/dl)	5.51 ± 1.95	4.44 ± 1.80	4.36 ± 1.38	**0.001**
iPTH (pg/ml)	270 (145-520)	161 (49-360)	196 (54-378)	**0.026**
Urea nitrogen (mg/dl)	136 (106-177)	89 (67-113)	88 (69-112)	**<0.001**
Creatinine (mg/dl)	8.4 (6.3-9.6)	6.6 (5.5-8.7)	6.9 (5.5-8.9)	0.080
Total cholesterol (mg/dl)	178 (152-230)	197 (155-241)	193 (166-228)	0.541
Triglyceride (mg/dl)	127 (97-244)	140 (99-223)	128 (88-189)	0.113
HDL (mg/dl)	33.3 (24.0-40.6)	41.4 (32.3-52.2)	47.2 (38.7-59.7)	**<0.001**
LDL (mg/dl)	104.0 (91.3-138.4)	111.4 (82.1-146.8)	112.1 (89.1-135.0)	0.914
Uric acid (mg/dl)	7.95 ± 2.70	6.71 ± 1.65	6.80 ± 1.60	**0.002**

Abbreviations: ALP, alkaline phosphatase; ALT, alanine aminotransferase; AST, aspartate aminotransferase; CVD, cardiovascular disease; HDL, high density lipoprotein; iPTH, intact parathyroid hormone; LDL, low density lipoprotein; mGFR, measured glomerular filtration rate; N/L, neutrophil to lymphocyte ratio; WBC, white blood cell.

The boldface indicated that *P* values less than 0.05 are considered statistically significant.
